# Photosynthetic performance in cyanobacteria with increased sulphide tolerance: an analysis comparing wild-type and experimentally derived strains

**DOI:** 10.1007/s11120-021-00882-8

**Published:** 2021-11-22

**Authors:** Elena Martín-Clemente, Ignacio J. Melero-Jiménez, Elena Bañares-España, Antonio Flores-Moya, María J. García-Sánchez

**Affiliations:** Departamento de Botánica y Fisiología Vegetal, Universidad de Málaga, Campus de Teatinos s/n, 29071 Málaga, Spain

**Keywords:** *Microcystis aeruginosa*, *Oscillatoria*, Cyanobacteria, Sulphide resistance, Photosynthesis

## Abstract

Sulphide is proposed to have influenced the evolution of primary stages of oxygenic photosynthesis in cyanobacteria. However, sulphide is toxic to most of the species of this phylum, except for some sulphide-tolerant species showing various sulphide-resistance mechanisms. In a previous study, we found that this tolerance can be induced by environmental sulphidic conditions, in which two experimentally derived strains with an enhanced tolerance to sulphide were obtained from *Microcystis aeruginosa,* a sensitive species*,* and *Oscillatoria*, a sulphide-tolerant genus. We have now analysed the photosynthetic performance of the wild-type and derived strains in the presence of sulphide to shed light on the characteristics underlying the increased tolerance. We checked whether the sulphide tolerance was a result of higher PSII sulphide resistance and/or the induction of sulphide-dependent anoxygenic photosynthesis. We observed that growth, maximum quantum yield, maximum electron transport rate and photosynthetic efficiency in the presence of sulphide were less affected in the derived strains compared to their wild-type counterparts. Nevertheless, in ^14^C photoincoporation assays, neither *Oscillatoria* nor *M. aeruginosa* exhibited anoxygenic photosynthesis using sulphide as an electron donor. On the other hand, the content of photosynthetic pigments in the derived strains was different to that observed in the wild-type strains. Thus, an enhanced PSII sulphide resistance appears to be behind the increased sulphide tolerance displayed by the experimentally derived strains, as observed in most natural sulphide-tolerant cyanobacterial strains. However, other changes in the photosynthetic machinery cannot be excluded.

## Introduction

Because oxygenic photosynthesis seems to have evolved in cyanobacteria-like organisms living in sulphide-rich waters (Canfield 1998; Battistuzzi et al. 2004), sulphide is proposed to have influenced the primary stages of the evolution of oxygenic photosynthesis. Despite being an abundant compound during these primary stages of evolution of cyanobacteria, sulphide is toxic for most present-day species of this phylum, and also to most organisms, because it disrupts aerobic metabolism by inhibition of cytochrome *c* oxidase (Beauchamp et al. 1984; Cooper and Brown 2008; Klatt et al. 2015a, b) and it also irreversibly affects the PSII-binding oxygen reaction centre, inhibiting oxygenic photosynthesis (Cohen et al. 1986; Miller and Bebout 2004). However, several taxa of extant cyanobacteria can be found in sulphide-rich environments, showing one or more adaptations that confer sulphide resistance (García-Pichel and Castenholz 1990; Miller and Bebout 2004). Cohen et al. (1986) classified cyanobacteria according to the PSI and PSII sulphide-tolerance level and the capacity for performing anoxygenic photosynthesis using H_2_S as an e^−^ donor: (i) cyanobacteria with sulphide-sensitive oxygenic photosynthesis; (ii) species with sulphide-resistant oxygenic photosynthesis; (iii) cyanobacteria with sulphide-resistant oxygenic photosynthesis, although more sensitive than type (ii), concurrent with the partial induction of sulphide-dependent anoxygenic photosynthesis; and (iv) cyanobacteria in which H_2_S-dependent anoxygenic photosynthesis replaces oxygenic photosynthesis at high sulphide concentrations. Most of the cyanobacteria belong to group (i), exhibiting sulphide-sensitive oxygenic photosynthesis (Cohen et al. 1986; Miller and Bebout 2004; Myers and Richardson 2009). The last three mechanisms, by which cyanobacteria can tolerate sulphide, are exceptional and not widespread within the group (Cohen et al. 1986; Miller and Bebout 2004; Myers and Richardson 2009). On the other hand, as gaseous H_2_S diffuses rapidly through biological membranes (Mathai et al. 2009), its toxic effects are ameliorated in some tolerant cyanobacteria through elimination by oxidation to sulphur (Stal 1995; Den Uyl et al. 2016). The HS^−^ form is as toxic as H_2_S (Olson and Straub 2016), and it is also known that one eubacterial species can exclude HS^−^ through a specific channel (Czyzewski and Wang 2012). Therefore, it is assumed that there can be a spectrum of cyanobacterial adaptations to sulphide (Dick et al. 2018). However, sulphide tolerance in cyanobacteria is not a trait found in closely related taxa, and it seems not to be constrained by phylogeny (Miller and Bebout 2004; Dick et al. 2018), since this trait is distributed throughout the phylum and it seems that it can be gained or lost relatively quickly as a response to changes in sulphide levels (Miller and Bebout 2004).

We recently employed an evolutionary experimental approach to study this phenomenon, using sulphide as a selective agent in two species of cyanobacteria with different sulphide tolerances. *Microcystis aeruginosa* (Kützing) Kützing is a sulphide-sensitive species isolated from a non-sulphureous freshwater reservoir and unable to grow at concentrations above 0.1 mM total sulphide (Martín-Clemente et al. 2019). *Oscillatoria* sp. was isolated from a natural spa with a total sulphide concentration around 0.2 mM (Reul et al. 2020), and it is capable of growing at up to 0.7–0.8 mM sulphide but unable to grow at 0.9 mM sulphide (Martín-Clemente et al. 2019). We performed a ratchet protocol, in which populations of both species were subjected to increasing sulphide levels during several tens of generations (i.e. along 3–4 months) to detect the maximum tolerance that could be achieved by both species (Martín-Clemente et al. 2019). We found that the derived experimental populations significantly increased the initial sulphide tolerance observed in their ancestors, as these derived populations of *M. aeruginosa* and *Oscillatoria* sp. were able to grow at 0.4 and 2 mM sulphide, respectively (Martín-Clemente et al. 2019). This experiment demonstrated that sulphide resistance can be gained as a response to changes in sulphide concentrations and that the level of resistance acquired was dependent on the sulphide level present in the strain´s original habitat. However, the ratchet protocol does not disentangle if the mechanism allowing the enhanced resistance of the derived populations is genetic, through the selection of new genetic variants (adaptation) or physiological (i.e. caused by acclimation). Nevertheless, this question can be analysed using a complementary experimental evolutionary design (Rouco et al. 2014; Melero-Jiménez et al. 2019, 2020). On the other hand, if the resistance is due to an adaptation process a physiological cost of the mutation conferring tolerance in terms of lower growth rates in the absence of the selective agent is expected (Lenski 1998). This effect has been always observed in microalgae and cyanobacteria mutant strains resistant to different selective agents (Costas et al. 2007; López-Rodas et al. 2008, 2011), including sulphide-resistant cyanobacteria strains which also showed a lower photosynthetic efficiency in the absence of sulphide, as well as changes in morphology and pigment content (Fernández-Arjona et al. 2013; Bañares-España et al. 2016).

On the other hand, studying the photosynthetic performance of the derived populations in the presence of sulphide would allow testing which photosynthetic characteristics explain the increase in tolerance i.e. whether the sulphide-resistant strains show a sulphide-tolerant oxygenic photosynthesis as well as the possibility that they use sulphide in anoxygenic photosynthesis. It is known that there is a relationship between the sulphide tolerance of PSII, estimated by Chl *a* fluorescence, and the sulphide concentrations in the environments where the cyanobacteria strains proliferate (Miller and Bebout 2004). This relationship could also be found in the ancestral and experimentally derived strains of *M. aeruginosa* and *Oscillatoria* sp. as they show a different degree of tolerance to sulphide. The presence of anoxygenic photosynthesis has been observed in cyanobacteria inhabiting environments where sulphide is present in the photic zone (Cohen et al. 1975a, b; Padan 1979; García-Pichel and Castenholz 1990; Klatt et al. 2015a), some of which belonging to the *Oscillatoria* genus. However, anoxygenic photosynthesis is not observed in all sulphide-rich environments, e.g. in cyanobacteria isolated from sulphide-rich microbial mats, most species have not shown sulphide-dependent anoxygenic photosynthesis (Myers and Richardson 2009), although it has been observed in a few cases (Hamilton et al. 2018).

The main objective of our study was to analyse the changes in photosynthetic performance of the derived, experimental strains, to check whether their increased sulphide tolerance could be due to a higher PSII sulphide resistance and/or the induction of sulphide-dependent photosynthesis, especially in *Oscillatoria*. For this purpose, the effect of sulphide on the maximum quantum yield of PSII and the electron transport rate under different sulphide concentrations was analysed and compared between derived and wild-type strains, as well as the effect of sulphide on their growth rates. Moreover, to test the presence of anoxygenic photosynthesis, a ^14^C photoincorporation experiment was performed. On the other hand, to shed more light on the process of tolerance acquisition, we analysed if adaptation or acclimation was the mechanism involved in this process and its possible physiological cost in terms of growth and photosynthetic rates.

## Methods

### Experimental strains and culture conditions

Experiments were performed with wild-type strains of *M. aeruginosa* (Ma1Vc) and *Oscillatoria* sp. (O1LH), and one strain derived from each of them, showing a higher tolerance to sulphide. Details about the isolation of the ancestral strains, culture conditions, as well as the evolutionary experimental approach (ratchet protocol) to obtain populations showing the highest sulphide tolerance, are described in Martín-Clemente et al. (2019). We selected two populations at the end of the ratchet experiment for a further characterization. Only one *Oscillatoria* sp. population was able to grow at 2 mM sulphide and was named strain O1R. From the 11 experimental populations of *M. aeruginosa* capable of growing at 0.4 mM sulphide, one was randomly selected and named as strain Ma1R.

The proportion of the different sulphide species in a solution (H_2_S, HS^−^ and S^2−^) depends on pH. At pH 7.2, the pH measured in La Hedionda spa (from where strain O1LH was isolated) and the media used to isolate the derived strains (Martín-Clemente et al. 2019), approximately 35% is H_2_S and 65% is HS^−^ (Howsley and Pearson 1979). Henceforth, we will refer to the total amount of sulphide species (total sulphide) as *sulphide*.

Both *M. aeruginosa* and *Oscillatoria* sp. wild-type and derived strains cultures were maintained in mid-log exponential growth (Cooper 1991) at 20 °C, with continuous irradiance of 50 μmol m^−2^ s^−1^. The culture medium was BG11-50% (pH 7.2) for strain Ma1Vc; however, strains Ma1R, O1LH and O1R were cultured in BG11-50% enriched with sulphide. For this purpose, the BG11-50% medium was buffered with HEPES, pH 7.2 (5 mM for strains Ma1R and O1LH, and 20 mM for strain O1R) to maintain a stable pH after sulphide additions. Strain Ma1R was maintained at 0.4 mM sulphide, the maximum concentration at which it was able to grow. Strain O1LH was maintained at 0.2 mM, the sulphide concentration of the spring from which it was isolated. Finally, although strain O1R was able to proliferate at 2 mM in the ratchet experiment (Martín-Clemente et al. 2019), it was maintained in the laboratory at 1 mM sulphide, because at higher concentrations, sulphur precipitates appeared rapidly in growth cultures (Martín-Clemente et al. 2019). Sulphide was added every day to the cultures from a Na_2_S-aqueous NaOH master stock solution (pH ~ 13, 210–240 mM) to maintain sulphide concentration as close as possible to the target concentrations.

### Effect of sulphide on the growth rate of the derived strains

To gain insight into the degree of tolerance of the derived strains, growth rates at increasing sulphide concentrations were analysed. The changes in acclimated growth rate (*m*) of strain Ma1R were measured in mid-log exponentially growing cultures according to Crow and Kimura (1970):

1 $$m = {\text{ log}}_{{\text{e}}} \left( {N_{{\text{t}}} /N_{0} } \right)/t,$$

where *N*_t_ and *N*_0_ are the cell number after *t* = 5 d and at the start of the experiment, respectively. Replicates (*n* = 5) of strain Ma1R with an initial cell density of 2.5 × 10^5^ cells mL^−1^ were placed in 15 mL tubes (Falcon™, BD Biosciences, MA, USA) containing 4 mL of BG11-50% buffered with 20 mM HEPES (pH 7.2), in a range of sulphide concentrations (from 0 to 0.8 mM). The number of Ma1R cells was directly counted using a haematocytometer.

Due to the filamentous nature of *Oscillatoria* sp., there was no possibility of counting single cells. Instead, the biomass present in the cultures was estimated by chlorophyll *a* (Chl *a*) content. Thus, the amount of Chl *a* at the start (Chl *a*_0_) and after *t* = 5 d (Chl *a*_t_) was used in Eq. 1, instead of *N*_0_ and *N*_t_. The Chl *a* concentration was determined according to Wellburn (1994), using *N*, *N*-dimethylformamide as a solvent. Four replicates of *Oscillatoria* (0.05 µg Chl *a* mL^−1^) were prepared in 50 mL tubes (Falcon™) containing 20 mL of the same culture medium with increasing sulphide concentrations (from 0 to 2 mM). This initial Chl *a* content was chosen because it was similar to the Chl *a* concentration in *M. aeruginosa* samples at the initial cell density used in this experiment. Because sulphur precipitation occurred at sulphide concentrations > 1 mM, interfering with light absorption, and stable pH was increasingly difficult to maintain (Martín-Clemente et al. 2019), growth rates were not tested above 2 mM. After 5 d of culture in the presence of sulphide, samples were centrifuged at 6850 g for 8 min, and the pellet was frozen for Chl *a* determination. Cultures of both strains were incubated at the same irradiance and temperature as stock cultures. Because sulphide disappears from the medium within few hours in the range of concentrations used (Martín-Clemente et al. 2019), this compound was added to cultures every day from the Na_2_S-aqueous NaOH master stock solution.

### Acclimation vs. adaptation as a tolerance mechanism

The ratchet protocol through which the resistant strains were isolated (Martín-Clemente et al. 2019) is not designed to elucidate the mechanism (acclimation vs. adaptation) allowing the increase in tolerance. Therefore, a complementary experiment (Rouco et al. 2014; Melero-Jiménez et al. 2019, 2020) was carried out with the derived strains. This protocol relies on the assumption that, at least in bacteria, the acclimation process could last as much as 2–3 generations (Bennett and Lenski 1997). After these number of generations, the acclimation would be lost in the population, and only the tolerance acquired through random mutations or adaptation could be maintained.

Five replicates of each strain were started with 6 × 10^5^ cells (strain Ma1R) or 5 mg fresh weight (FW) of strain O1R, quantified as indicated in Martín-Clemente et al. (2019). Cultures were grown in 20 mL of BG11-50% buffered with 20 mM HEPES in 50 ml ventilated cell-culture flasks. First, strain Ma1R was grown in the absence of the selective agent, that is, with no sulphide, and strain O1R was maintained under the standard conditions of the wild strain, that is, at 0.2 mM sulphide. After 7 d (~ 4 generations) in non-selective conditions, growth rates were quantified (*m*_1_) in each culture, and another five populations of each strain were started from them (using the same initial biomass per strain). In this second stage, populations were maintained for 7 d under the maximum sulphide concentration they tolerate (0.4 mM and 2 mM sulphide for strains Ma1R and O1R, respectively). After that growth rates (*m*_2_) were measured again.

From the comparison of *m*_1_ (growth rate at control, non-selective conditions) and *m*_2_ (growth rate at high sulphide levels), it can be inferred whether the maximum resistance is the result of acclimation or adaptation (Rouco et al. 2014; Melero-Jiménez et al. 2019, 2020). If growth rates are lower at high sulphide than in control conditions (*m*_1_ > *m*_2_), this is an indication that the tolerance to sulphide was reversible and was lost during the period of incubation in control conditions, i.e. it was due to acclimation. However, if *m*_1_ ~ *m*_2_, this is an indication that the tolerance was fixed in the population, and that the process involved in the increase of resistance would be the occurrence of spontaneous mutations, i.e. adaptation (although other mechanisms cannot be excluded).

### PSII performance and electron transport rate in the presence of sulphide

In order to quantify the sulphide impact on the performance of PSII in each strain, we followed the protocol of Miller and Bebout (2004), with some variations. These authors determined the effect of sulphide concentration on *F*_v_/*F*_m_ relative to that of the sulphide-free control of each analysed strain. This parameter, when measured with the standard analytical protocols in pulse–amplitude–modulation (PAM) devices, is a poor estimator of the maximum quantum yield of PSII in these organisms, as the correct determination of *F*_o_ and *F*_m_ requires specific protocols in cyanobacteria (Ogawa et al. 2017); however, it can be useful to compare the PSII activity among different strains and treatments. Taking this into account, a WATER-PAM (Walz, Effeltrich, Germany) fluorometer was used in a temperature-controlled room (20 °C). Three replicates per strain containing 0.1 µg Chl *a* mL^−1^ were incubated in 3 mL BG11-50% buffered with 20 mM HEPES in a range of sulphide concentrations (from 0 to 0.8 mM sulphide) for 45 min under an irradiance (*I*) of 50 μmol m^−2^ s^−1^ and then for 15 min in darkness. Then, samples were transferred to the WATER-PAM cuvette and, following the standard protocol, *F*_o_ and *F*_m_ (after an actinic light pulse was given) were determined. The maximum quantum yield of PSII (*F*_v_/*F*_m_) was calculated as follows:

2 $$F_{{\text{v}}} /F_{{\text{m}}} = \frac{{(F_{m} - F_{0} )}}{{F_{m} }},$$

where *F*_v_ is the variable fluorescence computed as the difference between *F*_m_ (maximal fluorescence) and *F*_0_ (minimum or basal fluorescence) of dark-adapted cells.

The electron transport rate (ETR) was estimated using the photochemical efficiency of PSII (Φ_PSII_) at different *I* values following Genty et al. (1989):

3 $${\text{ETR}} = I \times \Phi_{{{\text{PSII}}}} \times 0.{36} \times,a^*$$

where the constant 0.36 is used because it is assumed that only 36% of the photons that reach the cells are absorbed by the PSII in cyanobacteria (Johnsen and Sakshaug 1993), and a* as the absorptance of the culture, defined as the proportion of *I* absorbed by the sample (Korbe-Peinado et al. 2004):

4 $$a^* \, = { 1} - T,$$

where *T* is the transmittance, calculated as the ratio between the incident *I* and the irradiance transmitted through the culture. Measurements of a* were made at 20 °C in a glass cuvette. For this purpose, samples (*n* = 3) of each strain containing 0.1 μg Chl *a* mL^−1^ in 3 mL of BG11-50%, 20 mM HEPES, were exposed to an incident irradiance of 50 μmol m^−2^ s^−1^, using fluorescent lamps.

The data of ETR-*I* were fitted to the Michaelis–Menten equation:

5 $${\text{ETR}} = {\text{ETR}}_{{{\text{max}}}} \times \frac{{I{ }}}{{(I_{0.5} + I)}}$$

where ETR_max_ is the irradiance-saturated electron transport rate, *I* is the irradiance and *I*_0.5_ is the half-saturation irradiance. The photosynthetic efficiency (α^ETR^) was estimated as the slope from the linear fit of the four initial values of the ETR-*I* relationship. The fitting of the ETR-*I* data to the Michaelis–Menten equation was performed using the free software PAST ver. 4.04 (Hammer et al. 2001).

### ^14^C photoincorporation

We followed the protocol from Myers and Richardson (2009) with some modifications. Photosynthetic rate was measured as [^14^C] NaHCO_3_ photoincorporation under five different conditions: (a) light, aerobic, without sulphide (oxygenic photosynthesis); (b) light, anaerobic, with sulphide (sulphide-tolerant oxygenic photosynthesis); (c) light, anaerobic, with sulphide and DCMU (anoxygenic photosynthesis); (d) light, aerobic with DCMU (first control) and (e) darkness and aerobic (second control).

Cultures in exponential growth were prepared to a final concentration of 8 × 10^5^ cells mL^−1^ for strains Ma1Vc and Ma1R, and 0.12 µg Chl *a* mL^−1^ for strains O1LH and O1R and incubated overnight to be used for inoculation. The experiment was carried out in BG11-50% buffered with HEPES 5 mM (pH = 7.2) in 8 mL septum vials, three for each experimental condition. Treatments (b) and (c) were conducted in anaerobic conditions in order to avoid non-biological sulphide oxidation, so both medium and vials were purged with sterile N_2_ for 45 min prior to the addition of sulphide.

Five mL of non-bubbled or N_2_-bubbled medium were injected into their corresponding vials. A final concentration of 0.5 mM sulphide (stock 0.1 M Na_2_S·9H_2_O) was added to (b) and (c) vials. DCMU was supplemented to a final concentration of 10 µM in treatments (c) and (d). DCMU blocks the electron path from PSII to PSI; thus, oxygenic photosynthesis is deactivated. Under the hypothesis that derived strains could perform anoxygenic photosynthesis in the presence of DCMU, it would be using H_2_S as an electron donor to PSI through a quinone reductase (SQR) (Hamilton et al. 2018). The experiment started with the addition of 30 µL from a H^14^CO_3_^−^ 0.1 mCi mL^−1^ solution (3 µCi per vial) and 1 mL of culture to every vial. Dark samples were tightly sealed; then vials were incubated at a saturating irradiance of 250 μmol m^−2^ s^−1^, 20 °C with shaking for 2 h. After incubation, 1 mL of each culture was added to new vials containing 300 µL of 10 N formic acid to stop the fixation reaction, so the ^14^C uptake could be quantified. Then the medium was evaporated by placing these new vials in an oven at 90 °C for 24 h. Finally, ^14^C uptake was quantified by scintillation counting (Beckman LS 6500).

### Photosynthetic oxygen production in the absence of sulphide and pigment content

Photosynthetic rates in the absence of sulphide were measured in all strains using an Oxygraph system DW1/AD liquid-phase Electrode (Hansatech Instruments Ltd, UK), connected to a controlled temperature circulating bath at 20 °C. The medium used for measurements was 2 mL BG11-50% buffered with 20 mM HEPES (pH = 7.2). A cell density of 5 × 10^6^ cells mL^−1^ was used for the incubations of *M. aeruginosa* strains or 20 mg FW in the case of *Oscillatoria* sp. strains. Six replicates per strain were incubated for 15 min in darkness and then exposed to eight irradiance (*I*) levels (from 5 to 300 μmol photons m^−2^ s^−1^) for 5 min each. Irradiance was provided by fluorescent lamps (OSRAM L 18 W/865) and measured with a spherical quantum sensor (US-SQS/L, Walz, Germany), connected to a radiometer (LI-COR^®^LI-250A, Li-Cor Biosciences, Lincoln, Nebraska, USA). The Chl *a* content of the samples was determined as indicated below.

Data of net photosynthetic rate (NPR) as a function of *I* were fitted to the equation of Edwards and Walker (1983):

6 $${\text{NPR}} = {\text{NPR}}_{{{\text{max}}}} \times \frac{{(I{ }{-}{ }I_{{\text{c}}}) { }}}{{(I{ } + { }I_{0.5}) }}$$

where NPR_max_ is the maximum irradiance-saturated NPR; *I*_c_ is the irradiance-compensation point and *I*_0.5_ is the half-saturation irradiance. The initial slope from the linear regression of the four-first values of the NPR-*I* relationship was used as a proxy of the photosynthetic efficiency (α^NPR^). The fit was performed by using GraphPad software Prism version 7.00 for Windows.

For determination of the pigment content, four aliquots of each strain were centrifuged at 6850 *g* for 8 min at 4 °C to isolate cells from the culture, and the pellet was frozen at − 18 °C. The Chl *a* and total carotenoids (TC) quantification in both *M. aeruginosa* and *Oscillatoria* sp. strains were performed using *N*, *N*-dimethylformamide as solvent. In *Oscillatoria* sp. samples, a disruptor (Pobel, Madrid, Spain) was used to facilitate cell breakdown. The phycocyanin (PC) and phycoerythrin (PE) extraction were carried out by adding phosphate buffer (0.1 M, pH 6.5) to the frozen pellets. To ensure cell lysis, samples were subjected to three 10 s pulses at 50 W at intervals of 40 s using a sonicator (Vibra-Cell™), at 4 °C. After 24 h of cold (4 °C) and darkness incubation, samples were centrifuged again at 20,900 *g* for 15 min, and the supernatant was removed for absorbance measurements in a spectrophotometer (Selecta UV-2005, Spain). Quantification of Chl *a* and TC was performed following Wellburn (1994), and the equations proposed by Beer and Eshel (1985) were used to calculate PC and PE concentrations.

### Statistical analysis

Growth rate, photosynthetic parameters, carbon fixation rate and pigment content values were compared using a Student *t* test; the homogeneity of variances was previously checked with the Bartlett test. The analyses were performed with the free software PAST ver. 4.04 (Hammer et al. 2001).

## Results

### Sulphide tolerance of the derived strains and mechanism (adaptation v. acclimation) of acquisition

**Fig. 1:**
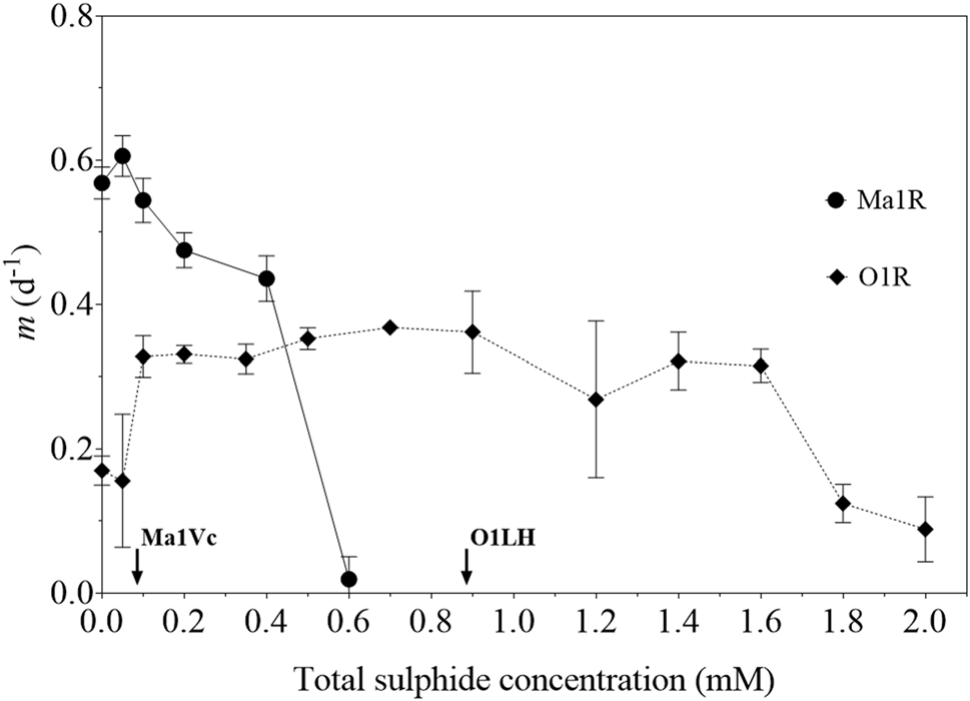
Effect of sulphide on the growth rate (*m*) of the derived strains of *Microcystis aeruginosa* (Ma1R) and *Oscillatoria* sp. (O1R). Growth rate was estimated in mid-log exponentially growing cultures by the increment in the number of cells in strain Ma1R, or the Chl *a* concentration in strain O1R, after 5 d of culture at increasing sulphide concentrations in the growth medium. The limit of tolerance of the wild-type strains (Ma1Vc and O1LH, Martín-Clemente et al. 2019), i.e. the concentration, i.e. the concentration at which growth was undetectable, are indicated on the X-axis. Data show mean ± SD (*n* = 5 for Ma1R; *n* = 4 for O1R)

The derived strain Ma1R was able to grow at high rates at sulphide concentrations above the limit of tolerance observed in its ancestor (0.1 mM; Martin-Clemente et al. 2019). Maximal growth was detected in 0.1 mM sulphide and growth rate declined slowly from this concentration up to 0.4 mM until it could be hardly detected in 0.6 mM sulphide, that is, almost sixfold the initial limit of tolerance of strain Ma1Vc (Fig. 1). The wild-type O1LH as well as the experimentally derived strain O1R exhibited a significantly higher sulphide tolerance compared to both *M. aeruginosa* strains (Fig. 1). Strain O1R showed significant growth in 2 mM sulphide, at least twice the O1LH limit of tolerance (Martín-Clemente et al. 2019). The *m* value of O1R was significantly higher (*P* < 0.01) when sulphide was present, showing maximal growth in the range 0.1–1.6 mM sulphide, decreasing progressively until 2 mM.

In order to disentangle whether this enhanced sulphide resistance was supported by acclimation or adaptation, strains Ma1R and O1R were cultivated first in control, non-selective conditions for several generations, and later, their tolerance was tested again by cultivation at high sulphide. There were not significant differences in the growth rate of strain Ma1R when grown without sulphide and in the presence of 0.4 mM sulphide (*P* > 0.05; Table 1). This result indicates that the tolerance was fixed in the population and points to adaptation as the mechanism involved in the increase of tolerance in this strain, although other mechanisms cannot be excluded. On the contrary, strain O1R grew at lower rates in 2 mM sulphide than in non-selective conditions (*m*_1_ > *m*_2_; Table 1), indicating that sulphide resistance was reversible, and then suggesting that the enhanced sulphide tolerance of this strain was due to an acclimation process.

**Table 1:** Growth rate (*m*; doublings · d^−1^) under non-selective conditions (0 and 0.2 mM sulphide for Ma1R and O1R, respectively; *m*_1_) and under the highest sulphide concentration tolerated by each strain (0.4 and 2 mM sulphide, respectively; *m*_2_)

	*m*_1_	*m*_2_	Tolerance mechanism
Ma1R	0.6 ± 0.04	0.6 ± 0.01	Adaptation
O1R	0.3 ± 0.02	0.1 ± 0.07*	Acclimation

Data are mean ± SD (*n* = 5)

*P < 0.001 (Student *t* test for comparison between strains of the same species)

### Effect of sulphide on *F*_v_/*F*_m_ and ETR-*I* relationship

**Fig. 2:**
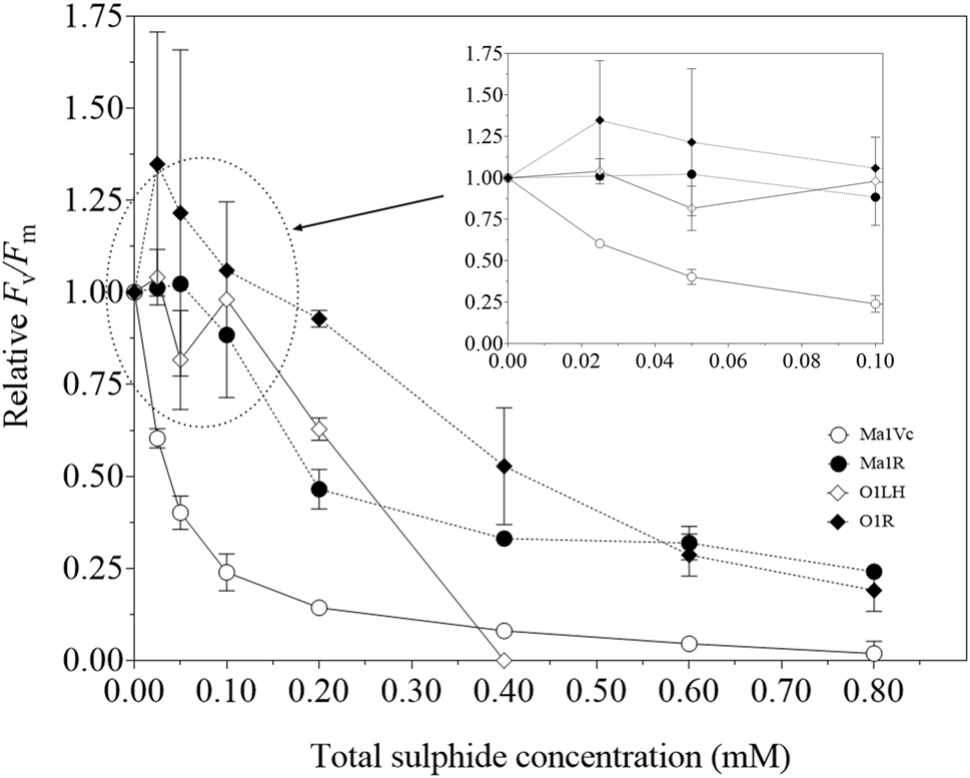
Effect of sulphide on maximum quantum yield from PSII (*F*_v_/*F*_m_), expressed as relative values with respect to control without sulphide, in wild-type (Ma1Vc, O1LH) and derived (Ma1R, O1R) strains of *M. aeruginosa* and *Oscillatoria*, respectively, after 1 h of exposure at increasing sulphide concentrations. Data show mean ± SD (*n* = 3)

In the absence of sulphide, *F*_v_/*F*_m_ values were 0.64 ± 0.02 for strain Ma1Vc, 0.58 ± 0.05 for Ma1R, 0.26 ± 0.03 for O1LH and 0.17 ± 0.04 for O1R (*n* = 3). Considering the differences between species, the data of *F*_v_/*F*_m_ were expressed as relative values with respect to their controls (i.e. in the absence of sulphide; Fig. 2). In concentrations ranging 0–0.1 mM sulphide (Fig. 2; inset), strains Ma1R, O1LH and O1R showed an invariant relative *F*_v_/*F*_m_ value. However, the values for Ma1Vc decreased 75% in this sulphide rank (Fig. 2; inset).

At 0.2 mM sulphide, both *Oscillatoria* strains exhibited higher relative *F*_v_/*F*_m_ values than *M. aeruginosa* strains (Fig. 2). However, at 0.4 mM sulphide, *F*_v_/*F*_m_ was undetectable in strain O1LH and was considered as 0 in relative value. At this concentration, strain O1R showed relative *F*_v_/*F*_m_ values higher than those displayed by strain Ma1R (Fig. 2). At ≥ 0.6 mM sulphide, no differences between the two derived strains were detected (Fig. 2).

**Fig. 3:**
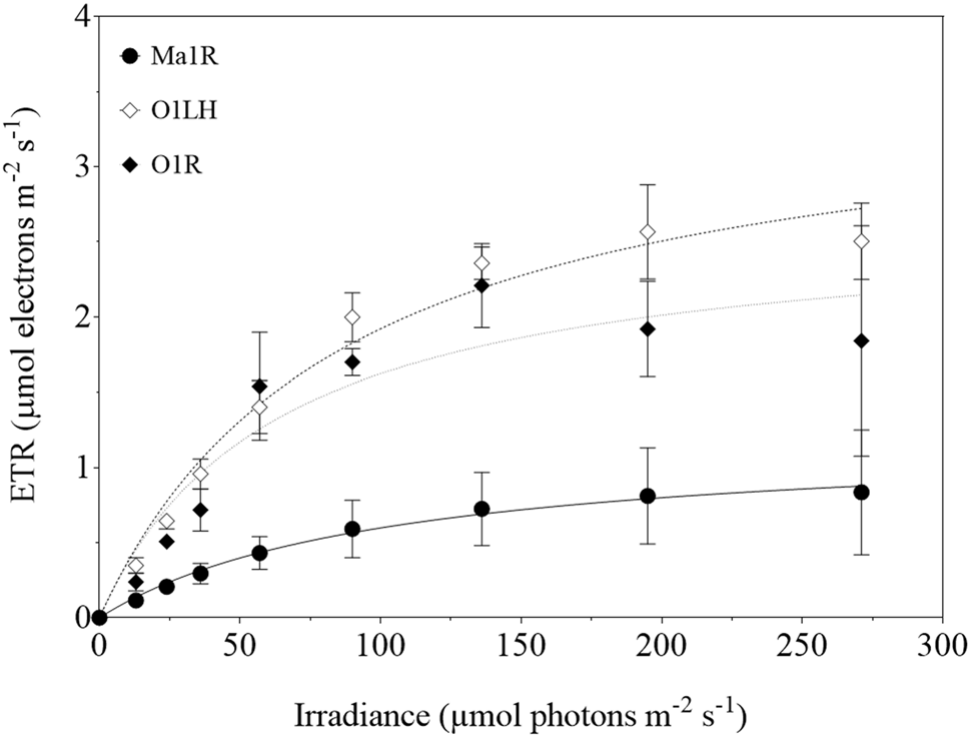
Electron transport rate (ETR) as a function of irradiance in *M. aeruginosa* derived (Ma1R; —•—) and *Oscillatoria* wild-type (O1LH; ---⋄---) and derived (O1R; ···♦···) strains in 0.2 mM sulphide. Data show mean ± SD (*n* = 3). Lines indicate the curve fitting to the Michaelis–Menten equation. The ETR of strain Ma1Vc was undetectable at this sulphide concentration

To analyse the effect of sulphide on the ETR as a function of *I*, the strains’ absorptance (a*) was firstly estimated (0.24 ± 0.02 and 0.12 ± 0.03 for strains Ma1Vc and Ma1R, and 0.27 ± 0.02 and 0.37 ± 0.01 for strains O1LH and O1R; *n* = 3). The reduction of the a* for Ma1R could be due to the lower Chl *a* content of this strain (Table 5). A ETR-*I* relationship was obtained at increasing sulphide concentrations, and the results observed at 0.2 mM sulphide are shown as an example in Fig. 3.

ETR_max_ and α^ETR^ values were computed for each strain by fitting ETR-*I* data at different sulphide concentrations (Table 2). In the absence of sulphide, ETR_max_ in strain Ma1Vc was roughly twice that of the derived strain Ma1R, although these strains did not show significant differences at 0.025 and 0.05 mM sulphide (Table 2). However, Ma1Vc ETR_max_ decreased more than 50% in this range, whereas the ETR_max_ of strain Ma1R remained quite constant. Furthermore, at 0.1 mM sulphide, the ETR_max_ of strain Ma1Vc decreased up to 80% compared to the control, whereas Ma1R values diminished by 30% (Table 2). At concentrations > 0.1 mM sulphide, ETR was not detected in strain Ma1Vc (Table 2; Fig. 3), whereas strain Ma1R experienced a progressive decrease in ETR_max_ values (Table 2). The α^ETR^ values followed the same tendency as ETR_max_, showing differences between strains only at 0 and 0.1 mM sulphide (Table 2). However, in the derived strain Ma1R, α^ETR^ values remained quite constant in the range 0–0.2 mM, whereas Ma1Vc values severely decreased when sulphide was added (Table 2).

**Table 2:** Effect of sulphide on the irradiance-saturated electron transport rate (ETR_max_) and photosynthetic efficiency (α^ETR^) in wild-type and derived strains of *M. aeruginosa* (Ma1Vc and Ma1R, respectively) and *Oscillatoria* sp. (O1LH and O1R, respectively)

Sulphide (mM)	*Microcystis aeruginosa*				*Oscillatoria sp.*			
	ETR_max_ (μmol e^−^ m^−2^ s^−1^)		α^ETR^ (mol e^−^ mol photons^−1^)		ETR_max_ (μmol e^−^ m^−2^ s^−1^)		α^ETR^ (mol e^−^ mol photons^−1^)	
	Ma1Vc	Ma1R	Ma1Vc	Ma1R	O1LH	O1R	O1LH	O1R
0	5.7 ± 0.4	3.0 ± 0.3**	0.39 ± 0.001	0.21 ± 0.02**	4.5 ± 0.1	4.2 ± 1.4	0.42 ± 0.03	0.29 ± 0.13
0.025	2.8 ± 0.3	3.1 ± 0.6	0.21 ± 0.02	0.21 ± 0.03	3.2 ± 0.3	2.5 ± 1.3	0.37 ± 0.00	0.26 ± 0.12
0.05	2.4 ± 0.4	2.9 ± 0.9	0.16 ± 0.001	0.19 ± 0.04	4.2 ± 1.9	2.3 ± 0.1	0.31 ± 0.06	0.17 ± 0.14
0.1	0.5 ± 0.4	2.1 ± 0.9*	0.05 ± 0.03	0.15 ± 0.03*	2.2 ± 0.2	2.4 ± 1.4	0.29 ± 0.02	0.3 ± 0.12
0.2	–	1.2 ± 0.7	–	0.1 ± 0.02	3.6 ± 0.4	2.5 ± 1.3	0.32 ± 0.03	0.25 ± 0.05
0.4	–	0.4 ± 0.4	–	0.04 ± 0.03	–	3.4 ± 1.9	–	0.28 ± 0.03
0.6	–	0.08 ± 0.05	–	0.01 ± 0.00	–	0.61 ± 0.71	–	0.06 ± 0.03
0.8	–	0.2 ± 0.1	–	0.02 ± 0.00	–	–	–	–

The values of ETR_max_ were computed by fitting of ETR-*I* data to the Michaelis–Menten model, whereas α^ETR^ values were computed as the slope from the linear fit of the four initial data of the ETR-*I* curve. Data show mean ± SD (*n* = 3). When *ETR* could not be detected, it is indicated by a hyphen

*P < 0.05; **P < 0.001 (Student *t* test for comparison between strains of the same species)

The ETR_max_ and α^ETR^ values of both *Oscillatoria* strains did not show significant differences at any sulphide concentration (Table 2; Fig. 3). Furthermore, ETR_max_ values in strain O1LH did not show a clear pattern of variation between 0.025 and 0.2 mM sulphide, because a decrease in the ETR values was observed, in some cases, at higher irradiances (data not shown). As indicated before, fluorescence was undetectable in 0.4 mM sulphide. In strain O1R, a decrease in ETR_max_ of ca. 40% in 0.025 mM sulphide was observed; however, from this concentration, values remained quite stable up to 0.2 mM, rising slightly in 0.4 mM and decreasing dramatically in 0.6 mM sulphide (Table 2) and, finally, ETR was not detected in 0.8 mM sulphide. Regarding α^ETR^, no statistically significant differences were detected between strains (Table 2). Although α^ETR^ decreased in 0.025 mM sulphide, O1LH efficiency values remained quite stable up to 0.2 mM sulphide. On the other hand, the α^ETR^ of strain O1R persisted relatively constant up to 0.4 mM sulphide (Table 2), decreasing in 0.6 mM sulphide.

### ^14^C photoincorporation

Neither *Oscillatoria* nor *M. aeruginosa* strains could carry out anoxygenic photosynthesis using H_2_S as an electron donor, since no significant carbon fixation was detected in 10 µM DCMU and 0.5 mM sulphide in all strains (Table 3).

**Table 3:** Carbon fixation rate (nmol C µg Chl *a*^−1^ h^−1^) of wild-type (Ma1Vc, O1LH) and derived (Ma1R, O1R) strains of *M. aeruginosa* and *Oscillatoria* sp., respectively, under light, aerobic; light, anaerobic plus 0.5 mM sulphide; light, anaerobic and 0.5 mM sulphide, plus 10 µM DCMU; light, anaerobic plus 10 µM DCMU; and darkness and aerobic conditions

Treatment	Carbon fixation rate (nmol ^14^C µg Chl *a*^−1^ h^−1^)			
	Ma1Vc	Ma1R	O1LH	O1R
No sulphide	160.2 ± 11.4	146.2 ± 8.2	116.5 ± 25.3	141.59 ± 28.7
Sulphide	1.3 ± 0.5	6.0 ± 1.9*	38.77 ± 6.77	43.76 ± 18.23
Sulphide + DCMU	0.1 ± 0.0	0.5 ± 0.5	0.03 ± 0.01	0.10 ± 0.03*
No sulphide + DCMU	1.2 ± 0.2	2.6 ± 0.7*	0.34 ± 0.03	0.74 ± 0.03***
Darkness	0.8 ± 0.3	1.6 ± 0.1**	0.36 ± 0.03	0.63 ± 0.26

Data are mean ± SD (*n* = 3)

*P < 0.05; **P < 0.01; ***P < 0.001 (Student *t* test for comparison between strains of the same species)

In the absence of sulphide, there were no significant differences in ^14^C photoincorporation between ancestral and derived populations of both species. In 0.5 mM sulphide, no resistant oxygenic photosynthesis was displayed in *M. aeruginosa* strains; photosynthesis was only about 1% (Ma1Vc) and 4% (Ma1R) of the control values in the absence of sulphide (Table 3). Nevertheless, higher sulphide tolerance of oxygenic photosynthesis was evident in *Oscillatoria* strains when exposed to 0.5 mM sulphide (Table 3). Specifically, the photosynthetic rate at 0.5 mM sulphide was approximately 30% of the control, in both strains (Table 3).

### Photosynthetic oxygen evolution vs. irradiance in the absence of sulphide

**Fig. 4:**
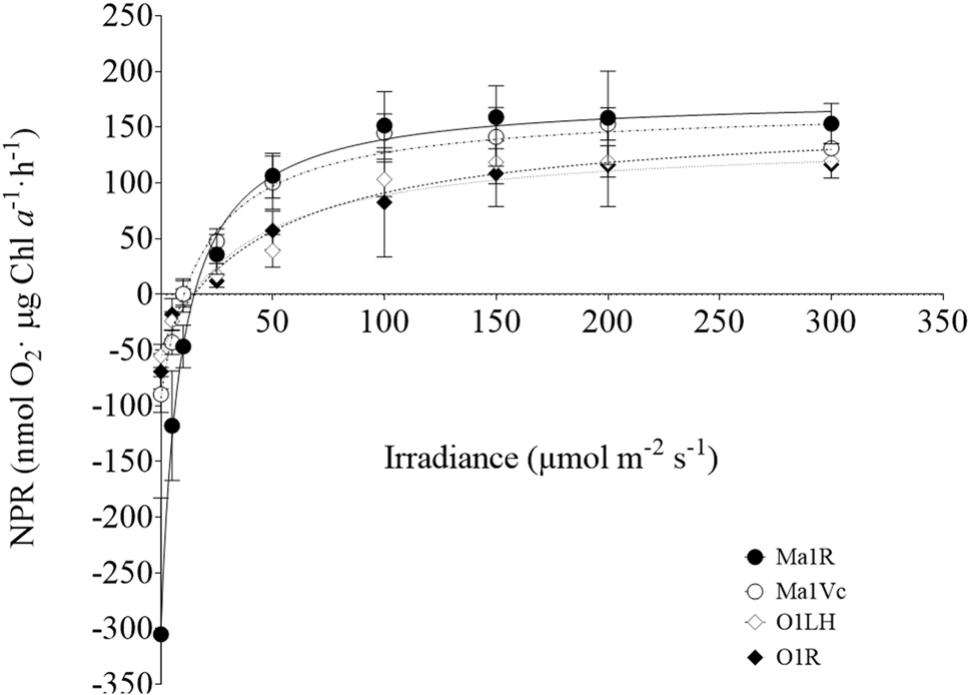
Net photosynthetic rates as a function of irradiance in *M. aeruginosa* wild-type (Ma1Vc; -^.^-○-^.^-) and derived (Ma1R; —•—) and *Oscillatoria* wild-type (O1LH; ---⋄---) and derived (O1R; ···♦···) strains in the absence of sulphide. Data show mean ± SD (*n* = 6). Lines indicate the curve fitting to the Edwards and Walker (1983) equation

There were no differences between ancestral and derived strains of both species when photosynthesis was measured as oxygen evolution in the absence of sulphide (Fig. 4). Maximum net photosynthetic rate (NPR_max_), compensation irradiance (*I*_c_), and photosynthetic efficiency (α^NPR^) were calculated (Table 4) from the NPR vs. irradiance curves (Fig. 4), not showing significant differences among strains of the same species except for *I*_c_ of *M. aeruginosa* strains, which was slightly higher in Ma1R than in Ma1Vc. However, when the NPR was referred to the number of cells, instead of Chl *a* content, in *M. aeruginosa* strains, the NPR_max_ of Ma1Vc was twice the value observed in strain Ma1R (63 ± 4 and 32 ± 1 nmol O_2_ µg 10^6^ cell^−1^ h^−1^, respectively), as the Chl *a* content was also twofold higher in strain Ma1Vc than in its derived strain (Table 5). There were not significant differences in dark respiration rates between *Oscillatoria* strains (51 ± 4 and 69 ± 12 nmol O_2_ µg Chl *a*^−1^ h^−1^ in O1LH and O1R, respectively; *P* > 0.05, Student *t* test) but respiration rates were higher in strain Ma1R than in the wild-type strain when referred both to Chl *a* content (− 305 ± 122 and − 90 ± 16 nmol O_2_ µg Chl *a*^−1^ h^−1^, respectively) or to number of cells (− 55 ± 18 and 35 ± 6 nmol O_2_ µg 10^6^ cell^−1^ h^−1^, respectively; *P* < 0,001, Student *t* test).

**Table 4:** Photosynthetic parameters derived from the fitting to the Edwards and Walker (1983) model of the NPR-*I* plots (Fig. 4) of wild-type (Ma1Vc; O1LH) and derived (Ma1R, O1R) strains of *M. aeruginosa* and *Oscillatoria*, respectively

	Ma1Vc	Ma1R	O1LH	O1R
NPR_max_ (nmol O_2_ µg Chl *a*^−1^ h^−1^)	168 ± 10	178 ± 6	160 ± 25	143 ± 36
*I*_c_ (µmol m^−2^ s^−1^)	10 ± 1	15 ± 1*	15 ± 4	14 ± 5
α^NPR^	2.0 ± 0.1	2.1 ± 0.6	2.7 ± 0.6	3.7 ± 1.0

The photosynthetic efficiency was computed as the slope from the linear fit of the four initial values

*P < 0.05; Student *t* test for comparison between strains of the same species

**Table 5:** Pigment content of ancestral (Ma1Vc, O1LH) and derived strains (Ma1R, O1R) of *M. aeruginosa* and *Oscillatoria*, respectively

	Ma1Vc	Ma1R	O1LH	O1R
	(μg 10^6^ cell^−1^)		(μg mg FW^−1^)	
[Chl *a*]	0.39 ± 0.03	0.19 ± 0.02**	0.17 ± 0.02	0.15 ± 0.01*
[TC]	0.13 ± 0.01	0.06 ± 0.01**	0.10 ± 0.02	0.05 ± 0.02**
[PC]	0.12 ± 0.02	0.10 ± 0.02	0.07 ± 0.03	0.01 ± 0.005*
[PE]	0.01 ± 0.001	0.09 ± 0.01**	0.07 ± 0.06	0.07 ± 0.07

Chl *a*, TC, PC and PE concentrations are expressed in μg 10^6^ cell^−1^ in *M. aeruginosa* strains, and in μg mg FW^−1^ in *Oscillatoria* strains. Data are mean ± SD (*n* = 4)

*P < 0.05; **P < 0.001; Student *t* test for comparison between strains of the same species

### Pigment content

There were significant differences between ancestral and derived *M. aeruginosa* strains in terms of Chl *a*, TC and PE contents, but not in the amount of PC (Table 5). Thus, the Chl *a* and TC contents in the derived strain Ma1R was half the amount observed in the wild type, whereas PE content was nine times higher in strain Ma1R than in strain Ma1Vc. Regarding *Oscillatoria* strains, Chl *a* was only slightly lower in the derived strain but TC also showed half the value of that measured in the wild-type strain. PC content was seven times lower in the derived strain O1R than in the wild type, but the amount of PE was similar in both strains.

## Discussion

Considering that the derived strains in this study were isolated from populations subjected for several generations to increasing high sulphide levels (Martín-Clemente et al. 2019), it was hypothesized that their growth and photosynthetic performance would be less affected in a sulphureous environment compared to their wild-type strain counterparts. This was in fact observed in the increased limit of tolerance to sulphide of the experimentally derived strains (Fig. 1). The high growth rates observed in strain O1R in the range 0.8–1.6 mM sulphide were consistent with the results found in other strains belonging to the genus *Oscillatoria*, which were isolated from springs containing 0.8 mM (13_1 strain) or 1.32 mM sulphide (U-Stink strain; Miller and Bebout 2004).

The parameter *F*_v_/*F*_m_ decreased at different rates in each strain with the increase of sulphide concentration (Fig. 2) and was indicative of differences in PSII efficiency in the presence of sulphide (Miller and Bebout 2004). The sulphide concentration required to reduce PSII performance by 50% can be used as an indication of the level of tolerance to this compound. Miller and Bebout (2004) compared the tolerance of PSII to H_2_S in twelve cyanobacterial strains isolated from different sulphureous springs, and they found a positive relationship between the PSII tolerance and the sulphide concentration of the environments where the strains were isolated. Similar results were observed in the present study, in experimentally derived and ancestral strains of cyanobacteria. The total sulphide dose required to induce a 50% PSII-inhibition was ~ 0.04 mM for strain Ma1Vc, ~ 0.2 mM for Ma1R, ~ 0.25 mM for O1LH and ~ 0.4 mM for O1R. The values observed in strains O1LH and O1R were higher than those observed in *Oscillatoria* strains DV-00-5 (0.12 mM) and DV-00-7 (0.06 mM, Miller and Bebout 2004). Those strains were isolated from environments with sulphide concentrations similar to that of La Hedionda (~ 0.2 mM): 0.29 and 0.17 mM sulphide, respectively (Miller and Bebout 2004). Furthermore, the 50% PSII-inhibition exhibited by strains O1LH and O1R was similar to those observed in *Oscillatoria* strains U-Stink (0.18 mM) and WHS-4 (0.3 mM), which were isolated from waters with much higher sulphide levels (1.3 and 4.9 mM sulphide, respectively, Miller and Bebout 2004). Therefore, strains O1LH and O1R possess a PSII resistant to relatively high sulphide concentrations, considering the sulphide levels measured in the water column of La Hedionda. However, sulphide concentrations could be higher inside the microbial mats from where strain O1LH was isolated, as it has been observed in these microenvironments (Klatt et al. 2016). On the other hand, it has to be taken into account that the protocol we used to measure the effect of sulphide on *F*_v_/*F*_m_ was slightly different from that used by Miller and Bebout (2004), so the 50% PSII-inhibition data could be not strictly compared.

Although strain O1LH showed a PSII sulphide tolerance higher than *M. aeruginosa* strains, at 0.4 mM sulphide, *F*_v_/*F*_m_ could not be detected in this *Oscillatoria* strain. This outcome may not be an indication of PSII total inhibition at this concentration because, in 0.5 mM sulphide, ^14^C photoincorporation by oxygenic photosynthesis was detected in this strain at a significant percentage of the control (around 30%; Table 3). On the other hand, despite measured at a higher time scale, growth rates of this strain were still high in this sulphide concentration (Martín-Clemente et al. 2019). Then, the undetectable value of *F*_v_/*F*_m_ could be explained by the low value of this parameter (0.26) shown by strain O1LH in the absence of sulphide. Despite being low, this value is similar to others already described in *Oscillatoria* (0.3–0.4, Ruangsomboon 2015; 0.15, Nath et al. 2017). Hence, the diminution of *F*_v_/*F*_m_ induced by sulphide additions could make this parameter impossible to detect at the highest sulphide concentrations.

ETR_max_ and α^ETR^ in both *Oscillatoria* strains, although affected by sulphide additions, did not decrease as much as in *M. aeruginosa* strains at concentrations > 0.1 mM sulphide (Table 2; Fig. 3). Although there is not always a direct relationship between electron flux and oxygen production (Masojídek et al. 2001), we could argue that oxygen production could be maintained in *Oscillatoria* strains at sulphide concentrations ranging from 0.1 to 0.4 mM, whereas, photosynthesis could already be inhibited in *M. aeruginosa* at these concentrations. In fact, carbon fixation by oxygenic photosynthesis was only partially inhibited at 0.5 mM sulphide in *Oscillatoria* strains but was almost completely inhibited in *M. aeruginosa* strains (Table 3). At higher sulphide concentrations, i.e. 0.6 mM sulphide, electron transport could still be observed in strain O1R, while being completely inhibited in both *M. aeruginosa* strains. These results would be consistent with the *m* values observed at this concentration: almost zero in strain Ma1R and basically unaltered in O1R cultures (Fig. 1). Nevertheless, we are aware that fluorescence and growth rate measurements are not strictly comparable.

Although it was hypothesized that strains O1LH and O1R could show anoxygenic photosynthesis as a tolerance mechanism as in some *Oscillatoria* strains (Cohen et al. 1986; Dods and Castenholz 1990), they did not show this trait. These results are similar to those obtained in representative members of the genera *Geitlerinema*, *Leptolyngbya* and *Oscillatoria* isolated from sulphide-rich environments (Richardson and Kuta 2003; Myers et al. 2007, Myers and Richarson 2009). On the other hand, in *M. aeruginosa*, the inability to perform anoxygenic photosynthesis was expected. In these strains, oxygenic photosynthesis was completely inhibited in 0.5 mM sulphide (Table 3), agreeing with the *F*_v_/*F*_m_ and complete inhibition of ETR in 0.4 mM sulphide in strain Ma1Vc and the considerable decrease in Ma1R cultures at this sulphide concentration (Fig. 2). Thus, we conclude that both *M. aeruginosa* strains show a PSII sensitive to low sulphide concentrations, being included in group (i) of Cohen et al. (1986), in which low sulphide concentrations (30–140 µM) block CO_2_ photoassimilation.

However, in 0.5 mM sulphide, both *Oscillatoria* strains were able to carry out oxygenic photosynthesis, although it was reduced by ~ 70% when compared to control values in the absence of sulphide. In this sense, strains O1LH and O1R displayed a partially resistant oxygenic photosynthesis which allows them to grow at this concentration (Martín-Clemente et al. 2019, Fig. 1). This partial resistance of oxygenic photosynthesis to sulphide recalls an *Oscillatoria terebriformis* population isolated from a sulphide spring, which was also described as a sulphide-resistant strain with no ability to perform anoxygenic photosynthesis (Castenholz 1977). Thus, both *Oscillatoria* strains, O1LH and O1R, could be included in group (ii) of Cohen et al. (1986), on the basis that oxygenic photosynthesis is still functioning due to PSII tolerance in 0.5–0.8 mM sulphide (pH 7.2), despite incapable of anoxygenic photosynthesis. This tolerance mechanism allows the wild-type strain O1LH to survive in La Hedionda spa, under moderate sulphide levels (0.1–0.3 mM) and oxygenated waters (Reul et al. 2020). However, the higher resistance shown by the derived strain O1R suggests that this *Oscillatoria* population could be able to survive if there were an increase in the sulphide concentration.

The tolerance of oxygenic photosynthesis to sulphide in cyanobacteria has been considered more important, under an ecological point of view, than the ability to use sulphide as an e^−^ donor, as well as being beneficial by providing additional oxygen for oxidative elimination of sulphide (Howsley and Pearson 1979; Stal 1995). Recent molecular studies have proposed that, taking into account that sulphide seems to attack D1 proteins in PSII, the replacement of alternative D1subunits among the four different types existing in cyanobacteria (Cardona et al. 2015) might confer tolerance to sulphide in this group (Dick et al. 2018). However, this hypothesis remains to be demonstrated as none of the characteristics observed by the four D1 types has been related to sulphide tolerance (Cardona et al. 2015) and because the known genome sequences of cyanobacteria strains showing sulphide-resistant oxygenic photosynthesis are very few (Dick et al. 2018).

While adaptation, i.e., the selection of new genetic variants, seems to be the mechanism allowing the enhanced tolerance in strain Ma1R, acclimation would be the process involved in the increase of tolerance in strain O1R. The lower growth rates in the absence of sulphide of strain Ma1R (Fig. 1) compared to the ancestral strain (Martín-Clemente et al. 2019) point out to the physiological cost of the genetic change conferring sulphide tolerance to strain Ma1R. This cost was also reflected in the lower photosynthetic efficiency (α^ETR^), ETR_max_ and NPR_max_ (when referred to number of cells) observed in Ma1R compared to Ma1Vc in this study. A similar response was also observed in a sulphide-resistant mutant derived from the Ma1G strain of *M. aeruginosa* (Bañares-España et al. 2016). In contrast, and as expected for an acclimation process, no significant differences were observed between strains O1LH and O1R in neither of these photosynthetic parameters nor in growth rates in the absence of sulphide (Martín-Clemente et al. 2019; Fig. 1).

The lower pigment content per cell in the experimentally derived strains, and specially of Chl *a* in strain Ma1R, points to a decrease in the number and/or size of photosynthetic units in sulphide-resistant cells. The decrease in Chl *a* content has been observed as a general response to stress (Tanaka and Tanaka 2006), as it is the presence of sulphide for a sensitive species. However, this effect was not found in the derived, sulphide-resistant Ma1G strain, where a conspicuous increase in Chl *a* was detected (Bañares-España et al. 2016). These results indicate that the increase in sulphide tolerance seems to involve a change not only on PS II performance but also at other levels of the photosynthetic machinery in both species. On the other hand, the high dark respiration rates observed in the sulphide-resistant strain of *M. aeruginosa* compared to its wild-type counterpart points to an increase in metabolism of the resistant strain that could be related with the trigger of mechanisms to cope with the stress imposed by sulphide, as observed with other environmental stressors in cyanobacteria (Mironov et al. 2019). In contrast, dark respiration rates were similar in O1LH and O1R, which would be in agreement with the fact that this species is already adapted to the presence of sulphide.

The analyses performed in this study allowed us to explore the photosynthetic characteristics that allow survival in the presence of sulphide by two representative cyanobacterial taxa, both in wild-type and derived (with increased sulphide resistance) strains, of which resistance relies on PSII performance, as observed in natural sulphide-tolerant cyanobacterial strains. Further studies, involving more cyanobacteria lineages, could clarify how sulphide tolerance has evolved in these microorganisms. Moreover, although it has been possible to shed some light on the PSII sulphide tolerance, we hypothesize that other physiological characteristics could be involved in the sulphide tolerance of the derived strains, which would require additional studies.

## Data Availability

Data and materials supporting the conclusions of the manuscript are available from the corresponding author on reasonable request.

## Abbreviations

*a**: Absorptance
Chl *a*: Chlorophyll *a*
ETR: Electron transport rate
ETR_max_: Irradiance-saturated electron transport rate
*F*_m_: Maximal fluorescence of dark-adapted cells
*F*_v_: Variable fluorescence of illuminated cells
*F*_0_: Basal fluorescence of dark-adapted cells
*F*_v_/*F*_m_: Maximum quantum yield of PSII
*I*: Irradiance
*I*_c_: Compensation irradiance
*m*: Growth rate
Ma1R: Derived strain of *Microcystis aeruginosa*
Ma1Vc: Wild-type strain of *Microcystis aeruginosa*
NPR: Net photosynthetic rate
O1LH: Wild-type strain of *Oscillatoria*
O1R: Derived strain of *Oscillatoria*
PC: Phycocyanin
PE: Phycoerythrin
PSII: Photosystem II
*T*: Transmittance
TC: Total carotenoids
α^ETR^: Photosynthetic efficiency calculated from ETR*-I* relationship
α^NPR^: Photosynthetic efficiency calculated from NPR-*I* relationship
Φ_PSII_: Photochemical efficiency of PSII
